# 6-Benzyl-3,4-dimeth­oxy-10-methyl­pyrido[2′,1′:2,3]imidazo[4,5-*c*]isoquinolin-5(6*H*)-one

**DOI:** 10.1107/S1600536809035806

**Published:** 2009-09-12

**Authors:** Kathrin Meindl, Daniel Stern, Fadime Mert-Balci, Uwe Beifuss

**Affiliations:** a Institut für Anorganische Chemie, Universität Göttingen, Tammannstrasse 4, 37077 Göttingen, Germany; b Bioorganische Chemie, Institut für Chemie, Universität Hohenheim, Garbenstrasse 30, 70599 Stuttgart, Germany

## Abstract

Pyrido[2′,1′:2,3]imidazo[4,5-*c*]isoquinolin-5(6*H*)-ones such as the title compound, C_24_H_21_N_3_O_3_, can be obtained in a few minutes in a microwave-assisted three-component reaction from 2-amino­pyridines, isocyanides and 2-carboxy­benz­aldehydes. In the title compound, the pyrido[2′,1′:2,3]imidazo[4,5-*c*]isoquinolin-5(6*H*)-one ring system is almost planar (mean deviation 0.068 Å). The dihedral angle between the benzyl ring and the pyrido[2′,1′:2,3]imidazo[4,5-*c*]isoquinolin-5(6*H*)-one ring system is 78.2°. The crystal structure is stabilized by inter­molecular C—H⋯O and C—H⋯N hydrogen bonds.

## Related literature

For the biological activity of fused imidazo[1,2-*a*]heterocycles, see: Almirante *et al.* (1965 ▶); Gueiffier *et al.* (1998 ▶); Sanfilippo *et al.* (1988 ▶); Varma & Kumar (1999 ▶). This heterocyclic structure element is present in drugs such as alpidem (anxiolytic), zolpidem (hypnotic) and zolimidine (anti­ulcer), see: Meng *et al.* (2007 ▶). For the synthesis of pyrido[2′,1′:2,3]imidazo[4,5-*c*]isoquinolin-5(6*H*)-ones by the microwave-assisted three-component reaction of 2-amino­pyridines, isocyanides and 2-carboxy­benzaldehydes, see: Mert-Balci *et al.* (2008 ▶).
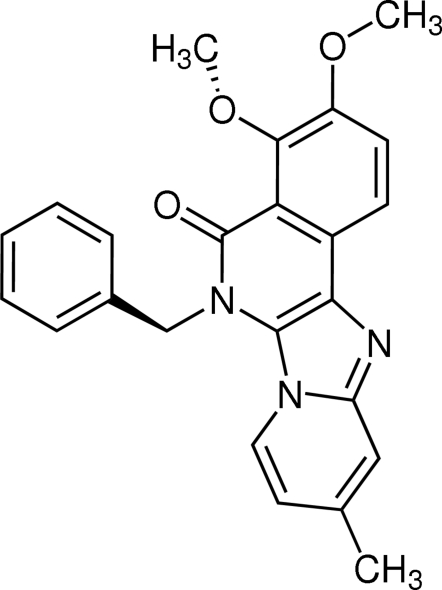


## Experimental

### 

#### Crystal data

C_24_H_21_N_3_O_3_
*M* *_r_* = 399.44
Monoclinic, 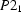

*a* = 8.3650 (17) Å
*b* = 7.0500 (14) Å
*c* = 15.983 (3) Å
β = 92.27 (3)°
*V* = 941.8 (3) Å^3^
*Z* = 2
Mo *K*α radiation
μ = 0.10 mm^−1^
*T* = 100 K
0.35 × 0.20 × 0.20 mm

#### Data collection

Bruker APEXII diffractometer
Absorption correction: multi-scan (*SADABS*; Bruker, 2006 ▶) *T*_min_ = 0.965, *T*_max_ = 0.981
16260 measured reflections
4479 independent reflections
4383 reflections with *I* > 2σ(*I*)
*R*_int_ = 0.021

#### Refinement

*R*[*F* ^2^ > 2σ(*F* ^2^)] = 0.030
*wR*(*F* ^2^) = 0.080
*S* = 1.06
4479 reflections
274 parameters
1 restraint
H-atom parameters constrained
Δρ_max_ = 0.23 e Å^−3^
Δρ_min_ = −0.20 e Å^−3^
Absolute structure: Flack (1983 ▶), 1895 Friedel pairs
Flack parameter: 0.2 (6)

### 

Data collection: *APEX2* (Bruker, 2006 ▶); cell refinement: *SAINT* (Bruker, 2006 ▶); data reduction: *SAINT*; program(s) used to solve structure: *SHELXS97* (Sheldrick, 2008 ▶); program(s) used to refine structure: *SHELXL97* (Sheldrick, 2008 ▶); molecular graphics: *SHELXTL* (Sheldrick, 2008 ▶); software used to prepare material for publication: *SHELXL97*.

## Supplementary Material

Crystal structure: contains datablocks global, I. DOI: 10.1107/S1600536809035806/lx2108sup1.cif

Structure factors: contains datablocks I. DOI: 10.1107/S1600536809035806/lx2108Isup2.hkl

Additional supplementary materials:  crystallographic information; 3D view; checkCIF report

## Figures and Tables

**Table 1:** Hydrogen-bond geometry (Å, °)

*D*—H⋯*A*	*D*—H	H⋯*A*	*D*⋯*A*	*D*—H⋯*A*
C5—H5*A*⋯N1^i^	0.95	2.68	3.4126 (16)	134
C18—H18*A*⋯O1^ii^	0.95	2.36	3.1127 (16)	136
C19—H19*A*⋯O1^iii^	0.95	2.58	3.3840 (16)	142

Symmetry codes: (i) 
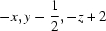
; (ii) 
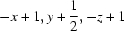
; (iii) 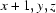.

**Fig. 1.**
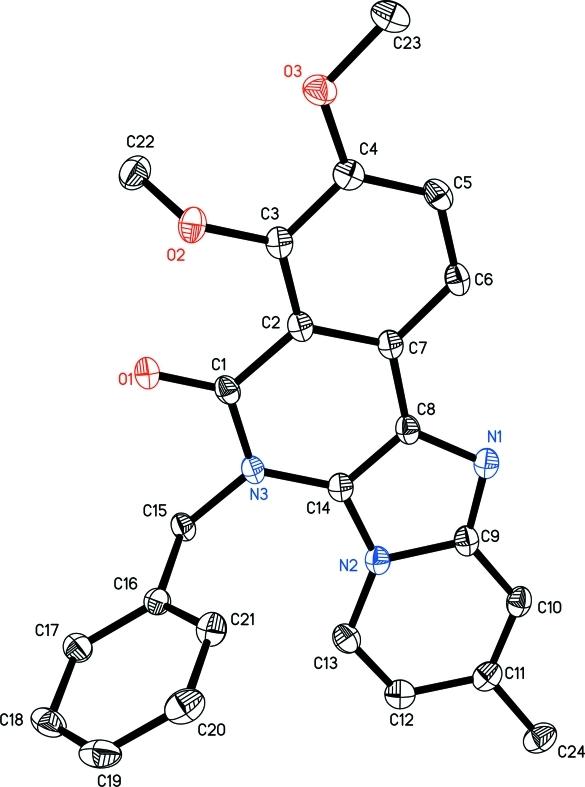
The molecular structure of the title compound, showing the atom-numbering scheme and 50% probability displacement ellipsoids. H atoms have been omitted for clarity.

**Fig. 2.**
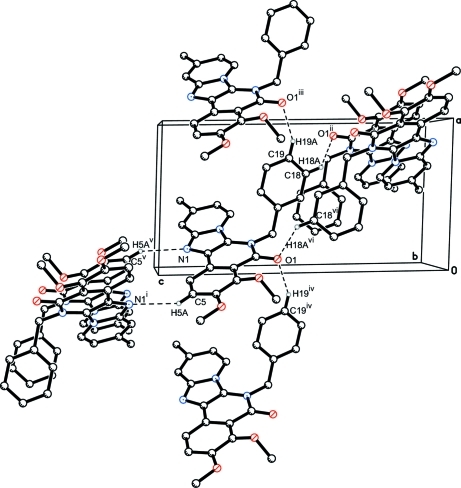
Hydrogen network in the crystal packing of the title compound. For clarity only H atoms involved in hydrogen bonds are shown. [Symmetry codes: (i) - x, y - 1/2, - z + 2; (ii) -x + 1, y + 1/2, - z + 1; (iii) x + 1, y, z; (iv) x - 1, y, z; (v) -x, y + 1/2, - z + 2; (vi) - x + 1, y - 1/2, - z + 1.]

**Fig. 3.**
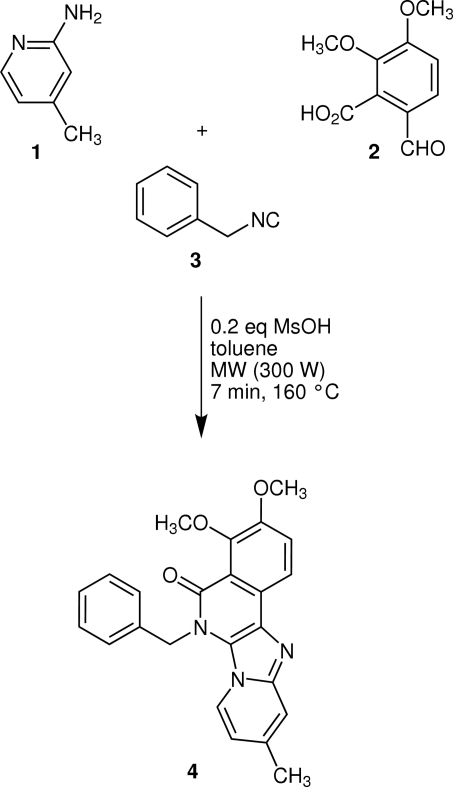
Microwave-assisted synthesis of the title compound.

## supplementary crystallographic information

### Comment

The fused imidazo[1,2-*a*]heterocycles have proven to be successful in the field of medicinal chemistry. They show important biological activities like antibacterial, antiviral, antifungal and anti-inflammatory properties (Gueiffier *et al.*, 1998, Almirante *et al.*, 1965, Varma & Kumar, 1999, Sanfilippo *et al.*, 1988). This heterocyclic structure element is present in drugs like alpidem (anxiolytic), zolpidem (hypnotic) and zolimidine (antiulcer) (Meng *et al.*, 2007). Recently, pyrido[2',1':2,3]imidazo[4,5-*c*]isoquinolin-5(6*H*)-ones, incorporating an imidazopyridine backbone, have been reported to exhibit potent antitumor activity *in vitro* (Meng *et al.*, 2007).

Pyrido[2',1':2,3]imidazo[4,5-*c*]isoquinolin-5(6*H*)-ones can be readily synthesized by the three-component reaction between 2-aminopyridines, isocyanides and 2-carboxybenzaldehydes under acidic conditions with the use of microwaves within a few minutes (Mert-Balci *et al.*, 2008). The title compound (Fig. 1) was obtained from the corresponding compounds under similar conditions (Fig. 3). The pyrido[2',1':2,3]imidazo[4,5-*c*]isoquinolin-5(6*H*)-one ring system is almost planar with a mean deviation from the plane of 0.0681 Å. The dihedral angle between the benzyl ring and the pyrido[2',1':2,3]imidazo[4,5-*c*]isoquinolin-5(6*H*)-one ring system is 78.2°. The molecules are hydrogen-bonded through hydrogen atoms at the benzyl carbon atoms C18 and C19, respectively, acting as donors towards the carbonyl oxygen atom O1 in different symmetry equivalent molecules, and by the hydrogen atom at C5 donating towards the imidazole nitrogen atom N1 (Fig. 2), thus forming a 3-dimensional network.

### Experimental

1 (1 mmol), 2 (1.09 mmol) and 3 (2.25 mmol) were suspended in toluene (2 ml) and placed in a 10 ml reaction vial that had been heated and cooled under argon. After the addition of MsOH (0.2 mmol), the vial was sealed with a septum and irradiated with microwaves (Discover by CEM; 2450 MHz; 300 W) at 160 °C for 7 min. The reaction mixture was allowed to cool to room temperature, diluted with CH_2_Cl_2_ (100 ml), and then washed with NaHCO_3_ solution (2 × 100 ml). The residue obtained after drying the organic phase over MgSO_4_ and concentration *in vacuo* was purified by column chromatography on silica gel (EtOAc) to yield the title compound 4 (yield 23%, m.p. 245–247°C). Crystallization from ethyl acetate provided suitable crystals of 4 for X-ray crystal structure analysis.

### Refinement

H atoms bonded to C atoms were placed at calculated positions and refined using a riding model. The constrained C—H distances were 0.95, 0.98 and 0.99 Å for aryl, methyl and methylene H atoms, respectively. The *U*_iso_(H) values were set at 1.5*U*_eq_(C) for methyl H atoms and 1.2*U*_eq_(C) for all other H atoms.

The absolute structure was confirmed by the method of Parsons' Q-values, which yielded an absolute structure parameter of 0.02 (18) (Parsons & Flack, 2004), and by a Hooft y parameter of -0.08 (16) (Hooft *et al.*, 2008).

### Crystal data

**Table:**

C_24_H_21_N_3_O_3_	*F*(000) = 420
*M**_r_* = 399.44	*D*_x_ = 1.408 Mg m^−^^3^
Monoclinic, *P*2_1_	Mo *K*α radiation, λ = 0.71073 Å
Hall symbol: P 2yb	Cell parameters from 6111 reflections
*a* = 8.3650 (17) Å	θ = 2.7–28.4°
*b* = 7.0500 (14) Å	µ = 0.10 mm^−^^1^
*c* = 15.983 (3) Å	*T* = 100 K
β = 92.27 (3)°	Block, orange
*V* = 941.8 (3) Å^3^	0.35 × 0.20 × 0.20 mm
*Z* = 2	

### Data collection

**Table:**

Bruker APEXII diffractometer	4479 independent reflections
Radiation source: fine-focus sealed tube	4383 reflections with *I* > 2σ(*I*)
graphite	*R*_int_ = 0.021
Detector resolution: 8.3333 pixels mm^-1^	θ_max_ = 28.5°, θ_min_ = 2.4°
ω scans	*h* = −10→11
Absorption correction: multi-scan (*SADABS*; Bruker, 2006)	*k* = −9→9
*T*_min_ = 0.965, *T*_max_ = 0.981	*l* = −21→21
16260 measured reflections	

### Refinement

**Table:**

Refinement on *F*^2^	Secondary atom site location: difference Fourier map
Least-squares matrix: full	Hydrogen site location: difference Fourier map
*R*[*F*^2^ > 2σ(*F*^2^)] = 0.030	H-atom parameters constrained
*wR*(*F*^2^) = 0.080	*w* = 1/[σ^2^(*F*_o_^2^) + (0.0521*P*)^2^ + 0.1311*P*] where *P* = (*F*_o_^2^ + 2*F*_c_^2^)/3
*S* = 1.06	(Δ/σ)_max_ < 0.001
4479 reflections	Δρ_max_ = 0.23 e Å^−^^3^
274 parameters	Δρ_min_ = −0.20 e Å^−^^3^
1 restraint	Absolute structure: Flack (1983)
Primary atom site location: structure-invariant direct methods	Flack parameter: 0.2 (6)

### Special details

**Table:**

Experimental. Intensities were measured with a Bruker *APEX* II area detector
Geometry. All e.s.d.'s (except the e.s.d. in the dihedral angle between two l.s. planes) are estimated using the full covariance matrix. The cell e.s.d.'s are taken into account individually in the estimation of e.s.d.'s in distances, angles and torsion angles; correlations between e.s.d.'s in cell parameters are only used when they are defined by crystal symmetry. An approximate (isotropic) treatment of cell e.s.d.'s is used for estimating e.s.d.'s involving l.s. planes.
Refinement. Refinement of *F*^2^ against ALL reflections. The weighted *R*-factor *wR* and goodness of fit *S* are based on *F*^2^, conventional *R*-factors *R* are based on *F*, with *F* set to zero for negative *F*^2^. The threshold expression of *F*^2^ > σ(*F*^2^) is used only for calculating *R*-factors(gt) *etc*. and is not relevant to the choice of reflections for refinement. *R*-factors based on *F*^2^ are statistically about twice as large as those based on *F*, and *R*- factors based on ALL data will be even larger.

### Fractional atomic coordinates and isotropic or equivalent isotropic displacement parameters (Å^2^)

**Table:**

	*x*	*y*	*z*	*U*_iso_*/*U*_eq_	
O1	0.10209 (10)	−0.19983 (13)	0.60571 (5)	0.02146 (17)	
O2	0.00203 (10)	−0.51480 (13)	0.68116 (5)	0.02068 (17)	
O3	−0.16121 (10)	−0.64479 (12)	0.80584 (5)	0.02256 (18)	
N1	0.16148 (11)	0.18429 (14)	0.89977 (6)	0.0188 (2)	
N2	0.28372 (11)	0.29948 (15)	0.78537 (6)	0.01719 (18)	
N3	0.19436 (11)	0.04583 (14)	0.68445 (5)	0.01692 (19)	
C1	0.11724 (12)	−0.12940 (17)	0.67556 (7)	0.0167 (2)	
C2	0.05157 (13)	−0.21455 (17)	0.75221 (6)	0.0163 (2)	
C3	−0.01653 (12)	−0.39733 (17)	0.74838 (6)	0.0174 (2)	
C4	−0.09563 (13)	−0.46934 (17)	0.81773 (7)	0.0187 (2)	
C5	−0.10004 (13)	−0.36386 (18)	0.89150 (7)	0.0196 (2)	
H5A	−0.1545	−0.4124	0.9379	0.024*	
C6	−0.02551 (13)	−0.18914 (17)	0.89725 (7)	0.0186 (2)	
H6A	−0.0260	−0.1206	0.9484	0.022*	
C7	0.05077 (12)	−0.11180 (17)	0.82860 (7)	0.0163 (2)	
C8	0.13440 (12)	0.06634 (16)	0.83200 (6)	0.0167 (2)	
C9	0.24986 (13)	0.32389 (17)	0.87093 (7)	0.0181 (2)	
C10	0.31098 (13)	0.48761 (18)	0.91234 (7)	0.0197 (2)	
H10A	0.2867	0.5093	0.9691	0.024*	
C11	0.40443 (13)	0.61520 (17)	0.87209 (7)	0.0210 (2)	
C12	0.44105 (14)	0.57844 (18)	0.78680 (8)	0.0221 (2)	
H12A	0.5087	0.6638	0.7588	0.027*	
C13	0.38171 (14)	0.42520 (17)	0.74504 (7)	0.0202 (2)	
H13A	0.4072	0.4041	0.6884	0.024*	
C14	0.20519 (13)	0.13373 (16)	0.76184 (7)	0.0169 (2)	
C15	0.25250 (13)	0.13309 (17)	0.60803 (6)	0.0176 (2)	
H15A	0.2373	0.2721	0.6112	0.021*	
H15B	0.1874	0.0860	0.5593	0.021*	
C16	0.42737 (13)	0.09168 (16)	0.59426 (7)	0.0168 (2)	
C17	0.48763 (14)	0.12660 (17)	0.51524 (7)	0.0199 (2)	
H17A	0.4187	0.1738	0.4714	0.024*	
C18	0.64788 (14)	0.09259 (18)	0.50067 (8)	0.0245 (2)	
H18A	0.6879	0.1175	0.4470	0.029*	
C19	0.74952 (14)	0.02273 (19)	0.56382 (9)	0.0269 (3)	
H19A	0.8587	−0.0014	0.5534	0.032*	
C20	0.69096 (15)	−0.0119 (2)	0.64258 (9)	0.0269 (3)	
H20A	0.7605	−0.0588	0.6863	0.032*	
C21	0.53069 (14)	0.02215 (17)	0.65755 (7)	0.0216 (2)	
H21A	0.4914	−0.0023	0.7114	0.026*	
C22	−0.13252 (15)	−0.51842 (19)	0.62248 (8)	0.0253 (2)	
H22A	−0.1096	−0.6038	0.5761	0.038*	
H22B	−0.1525	−0.3903	0.6007	0.038*	
H22C	−0.2273	−0.5636	0.6506	0.038*	
C23	−0.23837 (15)	−0.72755 (19)	0.87505 (8)	0.0253 (3)	
H23A	−0.2809	−0.8524	0.8589	0.038*	
H23B	−0.3263	−0.6454	0.8915	0.038*	
H23C	−0.1610	−0.7417	0.9223	0.038*	
C24	0.47030 (15)	0.7900 (2)	0.91476 (8)	0.0273 (3)	
H24A	0.4605	0.7778	0.9754	0.041*	
H24B	0.4101	0.9013	0.8946	0.041*	
H24C	0.5833	0.8050	0.9020	0.041*	

### Atomic displacement parameters (Å^2^)

**Table:**

	*U*^11^	*U*^22^	*U*^33^	*U*^12^	*U*^13^	*U*^23^
O1	0.0245 (4)	0.0245 (4)	0.0156 (4)	−0.0009 (3)	0.0033 (3)	−0.0029 (3)
O2	0.0243 (4)	0.0195 (4)	0.0182 (4)	0.0043 (3)	0.0001 (3)	−0.0029 (3)
O3	0.0264 (4)	0.0185 (4)	0.0230 (4)	−0.0020 (3)	0.0031 (3)	0.0031 (3)
N1	0.0191 (4)	0.0215 (5)	0.0156 (4)	0.0032 (4)	−0.0004 (3)	−0.0014 (4)
N2	0.0173 (4)	0.0184 (4)	0.0160 (4)	0.0033 (4)	0.0008 (3)	−0.0005 (4)
N3	0.0181 (4)	0.0199 (5)	0.0129 (4)	0.0010 (4)	0.0027 (3)	0.0003 (4)
C1	0.0141 (4)	0.0207 (5)	0.0152 (5)	0.0038 (4)	0.0025 (4)	0.0010 (4)
C2	0.0148 (4)	0.0196 (5)	0.0146 (5)	0.0047 (4)	0.0007 (4)	0.0011 (4)
C3	0.0161 (5)	0.0200 (5)	0.0159 (5)	0.0049 (4)	0.0000 (4)	0.0002 (4)
C4	0.0166 (5)	0.0188 (5)	0.0207 (5)	0.0030 (4)	−0.0007 (4)	0.0026 (4)
C5	0.0172 (5)	0.0239 (6)	0.0181 (5)	0.0037 (4)	0.0038 (4)	0.0038 (4)
C6	0.0193 (5)	0.0226 (5)	0.0142 (4)	0.0049 (4)	0.0022 (4)	0.0003 (4)
C7	0.0144 (5)	0.0197 (5)	0.0147 (4)	0.0040 (4)	0.0006 (4)	0.0006 (4)
C8	0.0156 (5)	0.0201 (5)	0.0143 (5)	0.0045 (4)	0.0012 (4)	−0.0001 (4)
C9	0.0174 (5)	0.0217 (6)	0.0152 (5)	0.0054 (4)	−0.0001 (4)	0.0002 (4)
C10	0.0191 (5)	0.0228 (6)	0.0171 (5)	0.0045 (4)	−0.0013 (4)	−0.0033 (4)
C11	0.0182 (5)	0.0198 (6)	0.0246 (5)	0.0045 (4)	−0.0028 (4)	−0.0030 (5)
C12	0.0210 (5)	0.0206 (6)	0.0246 (5)	0.0018 (4)	0.0006 (4)	0.0009 (4)
C13	0.0215 (5)	0.0196 (5)	0.0196 (5)	0.0022 (4)	0.0027 (4)	0.0020 (4)
C14	0.0166 (5)	0.0184 (5)	0.0156 (5)	0.0033 (4)	0.0003 (4)	0.0000 (4)
C15	0.0176 (5)	0.0215 (5)	0.0139 (4)	0.0018 (4)	0.0021 (4)	0.0020 (4)
C16	0.0174 (5)	0.0148 (5)	0.0182 (5)	−0.0001 (4)	0.0020 (4)	−0.0016 (4)
C17	0.0218 (5)	0.0170 (5)	0.0212 (5)	−0.0004 (4)	0.0037 (4)	−0.0002 (4)
C18	0.0253 (6)	0.0177 (5)	0.0313 (6)	−0.0036 (5)	0.0114 (5)	−0.0005 (5)
C19	0.0171 (5)	0.0208 (6)	0.0433 (7)	−0.0013 (4)	0.0058 (5)	−0.0043 (5)
C20	0.0216 (6)	0.0241 (6)	0.0346 (6)	0.0039 (5)	−0.0052 (5)	−0.0017 (5)
C21	0.0230 (5)	0.0211 (6)	0.0205 (5)	0.0031 (4)	−0.0004 (4)	−0.0011 (4)
C22	0.0304 (6)	0.0237 (6)	0.0214 (5)	−0.0029 (5)	−0.0042 (4)	−0.0009 (5)
C23	0.0236 (6)	0.0234 (6)	0.0290 (6)	−0.0007 (5)	0.0042 (5)	0.0065 (5)
C24	0.0243 (6)	0.0246 (6)	0.0327 (6)	−0.0004 (5)	−0.0018 (5)	−0.0076 (5)

### Geometric parameters (Å, °)

**Table:**

O1—C1	1.2238 (14)	C11—C24	1.5027 (17)
O2—C3	1.3702 (14)	C12—C13	1.3538 (17)
O2—C22	1.4364 (14)	C12—H12A	0.9500
O3—C4	1.3634 (14)	C13—H13A	0.9500
O3—C23	1.4273 (15)	C15—C16	1.5162 (15)
N1—C9	1.3246 (16)	C15—H15A	0.9900
N1—C8	1.3772 (14)	C15—H15B	0.9900
N2—C13	1.3838 (15)	C16—C21	1.3932 (16)
N2—C14	1.3852 (16)	C16—C17	1.4001 (16)
N2—C9	1.4180 (14)	C17—C18	1.3905 (16)
N3—C14	1.3833 (14)	C17—H17A	0.9500
N3—C1	1.3983 (15)	C18—C19	1.384 (2)
N3—C15	1.4675 (14)	C18—H18A	0.9500
C1—C2	1.4888 (15)	C19—C20	1.3905 (19)
C2—C3	1.4093 (17)	C19—H19A	0.9500
C2—C7	1.4198 (15)	C20—C21	1.3920 (17)
C3—C4	1.4080 (15)	C20—H20A	0.9500
C4—C5	1.3956 (16)	C21—H21A	0.9500
C5—C6	1.3820 (18)	C22—H22A	0.9800
C5—H5A	0.9500	C22—H22B	0.9800
C6—C7	1.4015 (16)	C22—H22C	0.9800
C6—H6A	0.9500	C23—H23A	0.9800
C7—C8	1.4376 (16)	C23—H23B	0.9800
C8—C14	1.3735 (15)	C23—H23C	0.9800
C9—C10	1.4160 (17)	C24—H24A	0.9800
C10—C11	1.3689 (18)	C24—H24B	0.9800
C10—H10A	0.9500	C24—H24C	0.9800
C11—C12	1.4325 (16)		
			
C3—O2—C22	114.36 (9)	N2—C13—H13A	120.2
C4—O3—C23	117.04 (10)	C8—C14—N3	124.06 (11)
C9—N1—C8	104.57 (9)	C8—C14—N2	106.58 (10)
C13—N2—C14	134.21 (10)	N3—C14—N2	129.36 (10)
C13—N2—C9	121.07 (10)	N3—C15—C16	113.18 (9)
C14—N2—C9	104.68 (9)	N3—C15—H15A	108.9
C14—N3—C1	120.01 (9)	C16—C15—H15A	108.9
C14—N3—C15	122.98 (10)	N3—C15—H15B	108.9
C1—N3—C15	116.91 (9)	C16—C15—H15B	108.9
O1—C1—N3	118.80 (10)	H15A—C15—H15B	107.8
O1—C1—C2	123.97 (11)	C21—C16—C17	118.79 (11)
N3—C1—C2	117.16 (9)	C21—C16—C15	122.45 (10)
C3—C2—C7	119.22 (10)	C17—C16—C15	118.75 (10)
C3—C2—C1	119.65 (10)	C18—C17—C16	120.31 (11)
C7—C2—C1	121.08 (10)	C18—C17—H17A	119.8
O2—C3—C4	118.08 (11)	C16—C17—H17A	119.8
O2—C3—C2	121.91 (10)	C19—C18—C17	120.50 (12)
C4—C3—C2	119.82 (10)	C19—C18—H18A	119.7
O3—C4—C5	125.26 (11)	C17—C18—H18A	119.7
O3—C4—C3	114.61 (10)	C18—C19—C20	119.63 (11)
C5—C4—C3	120.13 (11)	C18—C19—H19A	120.2
C6—C5—C4	120.28 (11)	C20—C19—H19A	120.2
C6—C5—H5A	119.9	C19—C20—C21	120.10 (12)
C4—C5—H5A	119.9	C19—C20—H20A	119.9
C5—C6—C7	120.85 (10)	C21—C20—H20A	119.9
C5—C6—H6A	119.6	C20—C21—C16	120.65 (11)
C7—C6—H6A	119.6	C20—C21—H21A	119.7
C6—C7—C2	119.51 (11)	C16—C21—H21A	119.7
C6—C7—C8	123.11 (10)	O2—C22—H22A	109.5
C2—C7—C8	117.35 (10)	O2—C22—H22B	109.5
C14—C8—N1	111.75 (10)	H22A—C22—H22B	109.5
C14—C8—C7	119.75 (10)	O2—C22—H22C	109.5
N1—C8—C7	128.45 (10)	H22A—C22—H22C	109.5
N1—C9—C10	129.72 (10)	H22B—C22—H22C	109.5
N1—C9—N2	112.40 (10)	O3—C23—H23A	109.5
C10—C9—N2	117.88 (10)	O3—C23—H23B	109.5
C11—C10—C9	121.21 (10)	H23A—C23—H23B	109.5
C11—C10—H10A	119.4	O3—C23—H23C	109.5
C9—C10—H10A	119.4	H23A—C23—H23C	109.5
C10—C11—C12	118.42 (11)	H23B—C23—H23C	109.5
C10—C11—C24	122.11 (11)	C11—C24—H24A	109.5
C12—C11—C24	119.46 (11)	C11—C24—H24B	109.5
C13—C12—C11	121.70 (12)	H24A—C24—H24B	109.5
C13—C12—H12A	119.2	C11—C24—H24C	109.5
C11—C12—H12A	119.2	H24A—C24—H24C	109.5
C12—C13—N2	119.60 (11)	H24B—C24—H24C	109.5
C12—C13—H13A	120.2		

### Hydrogen-bond geometry (Å, °)

**Table:**

*D*—H···*A*	*D*—H	H···*A*	*D*···*A*	*D*—H···*A*
C5—H5A···N1^i^	0.95	2.68	3.4126 (16)	134
C18—H18A···O1^ii^	0.95	2.36	3.1127 (16)	136
C19—H19A···O1^iii^	0.95	2.58	3.3840 (16)	142

Symmetry codes: (i) −*x*, *y*−1/2, −*z*+2; (ii) −*x*+1, *y*+1/2, −*z*+1; (iii) *x*+1, *y*, *z*.
